# Enhanced survival of hypoimmunogenic otic progenitors following intracochlear xenotransplantation: repercussions for stem cell therapy in hearing loss models

**DOI:** 10.1186/s13287-023-03304-9

**Published:** 2023-04-12

**Authors:** Luisa H. Andrade da Silva, Rachel A. Heuer, Christian B. Roque, Tammy L. McGuire, Tomonori Hosoya, Hironobu Kimura, Kouichi Tamura, Akihiro J. Matsuoka

**Affiliations:** 1 Department of Otolaryngology and Head and Neck Surgery, Feinberg School of Medicine, Northwestern University, Chicago, IL USA; 2 Department of Neurology, Feinberg School of Medicine, Northwestern University, Chicago, IL USA; 3 Kobe Research Institute, HEALIOS K.K., Kobe, Hyogo Japan; 4 Roxelyn and Richard Pepper Department of Communication Sciences and Disorders, School of Communication, Northwestern University, Evanston, IL USA; 5 Hugh Knowles Center for Clinical and Basic Science in Hearing and Its Disorders, Evanston, IL USA; 6 Center for Advanced Regenerative Engineering, Northwestern University, Evanston, IL USA; 7 Department of Otolaryngology and Head and Neck Surgery, University of California San Diego, 9444 Medical Center Drive, MC7895, La Jolla, CA 92037 USA

**Keywords:** Inner ear regeneration, Stem cell transplantation, Xenorejection, Gene engineering, Hypoimmunogenic derivatives

## Abstract

Stem cell replacement holds the potential for sensorineural hearing loss (SNHL) treatment. However, its translation into clinical practice requires strategies for improving stem cell survival following intracochlear transplantation. Considering recent findings showing that the inner ear contains a resident population of immune cells, we hypothesized that immune evasion would improve the survival and residence time of transplanted stem cells in the cochlea, potentially leading to better outcomes. To test this, we leveraged genetic engineering techniques to develop hypoimmunogenic human-induced pluripotent stem cells (hi-iPSC), which lack human leukocyte antigen expression. We found that gene editing does not affect the biological properties of hi-iPSCs, including their capacity to differentiate into otic neural progenitors (ONPs). Compared to wild-type ONPs, more hypoimmunogenic ONPs (derived from hi-iPSCs) were found in the inner ear of immunocompetent mice ten days following cochlear xenotransplantation. This approach may open a new avenue for experimental and clinical SNHL treatments.

**Supplementary Information:**

The online version contains supplementary material available at 10.1186/s13287-023-03304-9.

## Introduction

Sensorineural hearing loss (SNHL) is an irreversible auditory disorder that affects millions of people worldwide [1]. Factors such as aging, acoustic trauma, or exposure to ototoxins provoke the death of hair cells (HC) and/or degeneration of spiral ganglion neurons (SGN) [2, 3]. Given that these cells are not endowed with regenerative potential, these events lead to permanent hearing loss [4]. Accordingly, attention has been given to cell replacement approaches, in which exogenous stem cell derivatives, such as otic epithelial progenitors[5] and otic neural progenitors (ONPs) [6], are transplanted into the inner ear to replace lost cells. Overall, the efficacy of replacement therapy in preclinical models in SNHL studies has not been satisfactory due to donor cell death, limited engraftment in the host tissues, and poor connection with the host’s neural circuits [7].

In this context, bioengineering-based approaches have been explored to overcome the physiological and anatomical barriers of the inner ear that limit donor cell survival, distribution, and function [8]. A factor that remains unaddressed, however, is the recently reported cochlear immune cell population, which may orchestrate innate/adaptative responses to transplanted cells [9, 10]. Such responses may be even more impactful in xenotransplantation studies, that is, when cells of human origin are transplanted into animal models of SHNL. In fact, one week after intracochlear transplantation in guinea pigs, neuronal-like cells derived from human pluripotent stem cells (PSCs) were found surrounded by the host’s CD45^+^ leukocytes [11], and little is known about how these responses could affect the outcomes and reliability of preclinical studies findings. Although immunosuppressant drugs prevent immune response to transplanted cells, their use could be infeasible in the long term due to systemic adverse effects and susceptibility to opportunistic infections [12].

Recently, gene editing techniques have been explored to generate human-induced pluripotent stem cells with reduced immunogenicity (hi-iPSCs), looking into preventing their clearance by the host’s immune system and thus optimizing allogenic stem cell transplantation approaches [13]. Specifically, the hi-iPSCs are engineered to disrupt the surface expression of human leukocyte antigens (HLA) and to overexpress immune-suppressive molecules [13]. Importantly, the derivatives of hi-iPSCs retain the low-immunogenicity features, evade the recipient’s innate and adaptative responses, and exhibit prolonged survival after transplantation, even without the use of immunosuppressants [14–17]. Therefore, in the present work, we sought to investigate the feasibility of generating hypoimmunogenic ONPs (hi-ONP) from the hi-iPSCs. We also hypothesized that the immune evasion of hi-ONPs would lead to improved survival following intracochlear xenotransplantation, compared to wild-type cells. If feasible, this strategy could be explored in the future to facilitate the replacement of damaged SGNs by the donor ONPs, improving inner ear regeneration in preclinical and clinical SHNL studies.

## Results

### Genetic modifications do not affect the biological properties of hi-iPSCs

**Fig. 1:**
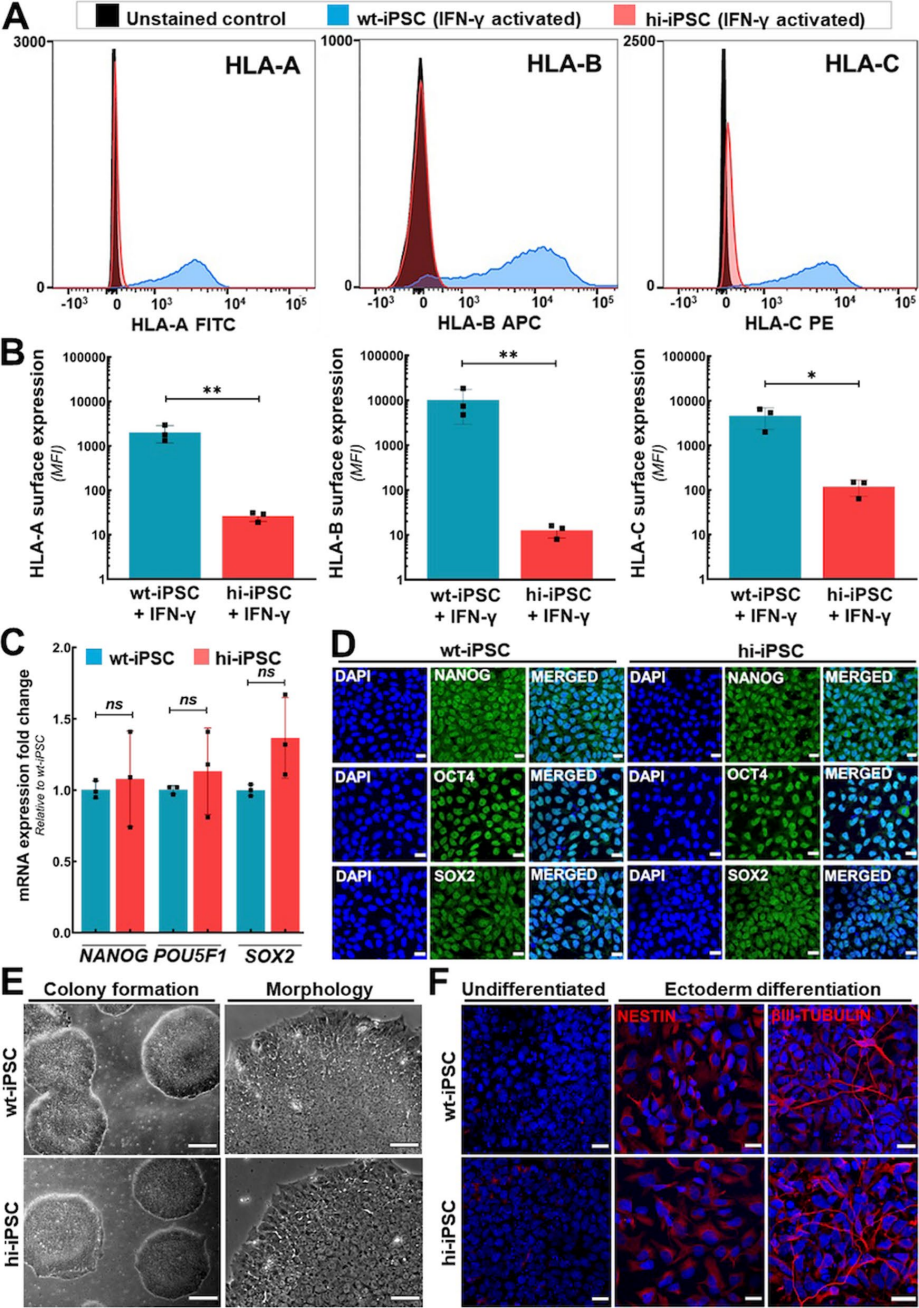
Hypoimmunogenic iPSC characterization. **A** Flow cytometry analysis. Note the absence of surface expression of HLA-A, HLA-B, and HLA-C molecules in hi-iPSCs (red) following stimulation with IFN-γ when compared to wild-type iPSCs (wt-iPSCs, blue). Mean fluorescent intensity values are shown in (**B**). Bars represent means ± standard deviation from three independent experiments. *p < 0.05, **p < 0.01. **C**, **D** Expression of pluripotency markers. **C** Quantification of *NANOG*, *POU5F1* (*OCT4*), and *SOX-2* mRNA levels by RT-qPCR analysis. Data show the average and standard deviation of fold change values from three independent experiments (ns: non-significant). **D** Representative immunocytochemistry photomicrographs evidencing Nanog, Oct4, and SOX2. Bars 20 μm. **E** Representative phase-contrast photomicrographs of iPSC cultures, evidencing unaltered morphology and colony formation capacity in hi-iPSCs when compared to wt-iPSCs. “Colony formation” bars 300 μm, “Morphology” bars 60 μm. **F** Differentiation assay into ectoderm germ layer. Representative immunocytochemistry photomicrographs of cells differentiated from both wt-iPSCs and hi-iPSCs, expressing the ectoderm markers Nestin and Beta-III Tubulin, which are absent in undifferentiated cells. Bars 20 μm

We performed genomic editing technology-mediated deletion of major HLA Class I genes to generate human iPSCs with reduced immunogenicity (hi-iPSC). Flow cytometry analysis data confirmed the effectiveness of genetic ablation, indicating significantly reduced surface expression of HLA-A, HLA-B, and HLA-C molecules in hi-iPSCs when compared to wild-type (wt) iPSCs (Fig. 1A, B). The hi-iPSCs were additionally modified to overexpress the immunomodulatory molecules HLA-G and programmed death ligand-1 (PD-L1), which target immune surveillance by NK cells and T cells, respectively. Compared to wt-iPSCs, the hi-iPSCs have significantly higher mRNA levels (Additional file 1: Fig. S1A) and surface expression of these immunomodulatory factors (Additional file 1: Fig. S1B, C). These genomic modifications did not provoke karyotype alterations in hi-iPSCs (Additional file 1: Fig. S2). When compared to wt-iPSCs, the mRNA levels (Fig. 1C) and protein expression (Fig. 1D) of pluripotent stem cell markers Nanog, Oct4, and SOX2 remained unaltered in hi-iPSCs. The hi-iPSCs also exhibited preserved colony formation capacity and unchanged morphology (Fig. 1E). Finally, the genomic modifications did not impair the capacity of hi-iPSCs to differentiate into advanced derivatives of the ectoderm germ layer (Fig. 1F). After directed differentiation, hi-iPSC derivatives were positive for the ectoderm markers Nestin and β-III Tubulin, similar to wt-iPSC derivatives.

### Generation of otic neuronal progenitors with reduced immunogenicity from hi-iPSCs

**Fig. 2:**
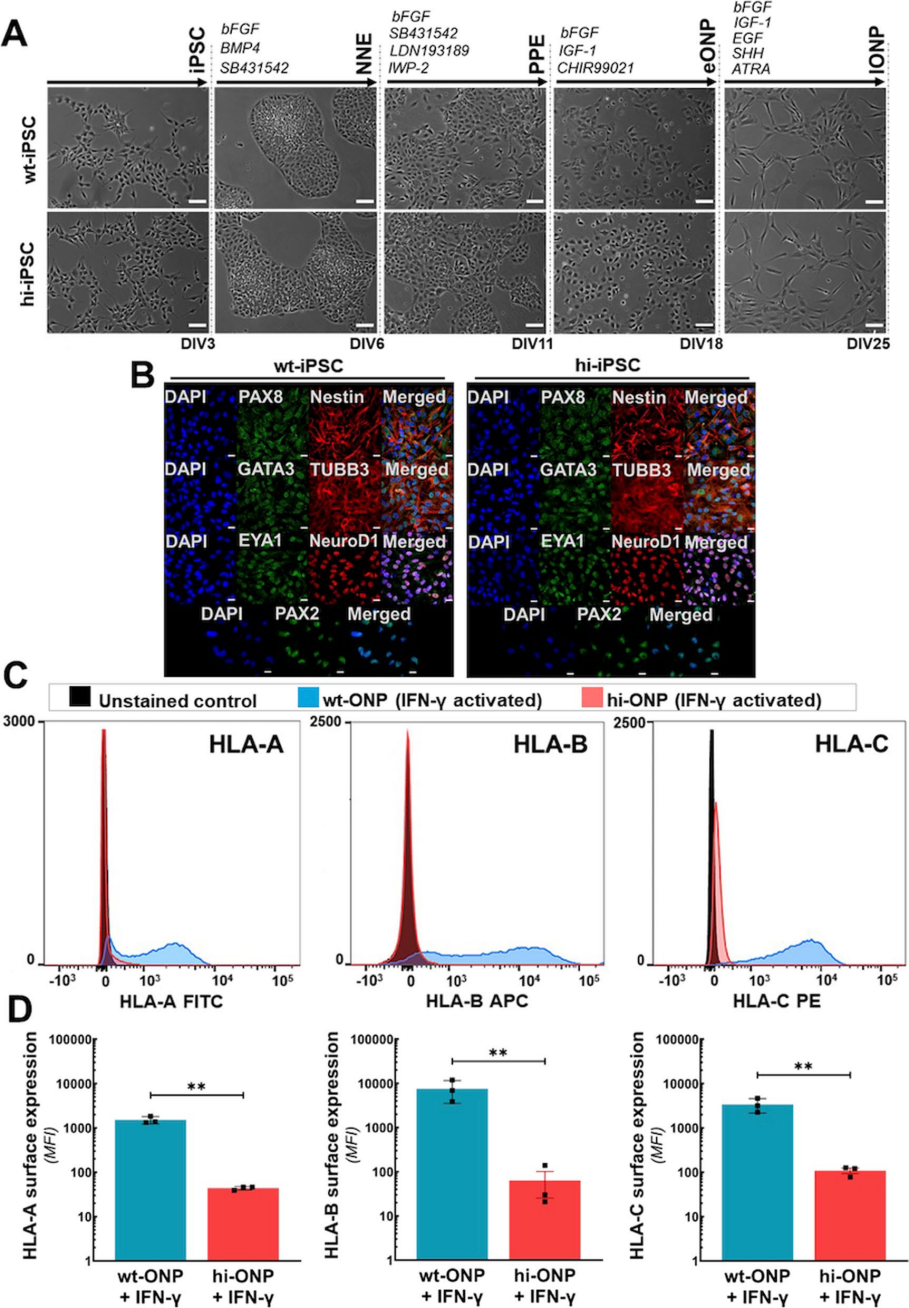
iPSC differentiation toward otic neuronal progenitors (ONPs). **A** Representative phase-contrast photomicrographs of differentiation steps from iPSC toward ONP. Note that hi-iPSCs exhibit the same morphological changes during differentiation when compared to wt-iPSCs. Bars 100 μm. NNE: Non-neuronal ectoderm; PPE: pre-placodal ectoderm; eONP: early-stage ONP; lONP: late-stage ONP. **B** Representative immunocytochemistry photomicrographs of iPSC-derived lONPs, evidencing expression of the neuronal markers Nestin, β-III Tubulin (TUBB3), and neurogenic differentiation 1 (NeuroD1). The images also show expression of the otic markers PAX8, GATA-3, EYA-1, and PAX-2. Bars 20 μm. **C**, **D** Flow cytometry analysis. **C** ONPs derived from hi-iPSCs (hi-ONP, red) continue to lack surface expression of HLA-A, HLA-B, and HLA-C molecules following stimulation with IFN-γ when compared to wild-type cells (wt-ONP, blue). Mean fluorescent intensity values are shown in (**D**). Bars represent means ± standard deviation from three independent experiments. **p < 0.01

After confirming that the hi-iPSCs committed to the ectoderm lineage, we differentiated them into late-stage ONPs. The hi-iPSCs were sequentially differentiated into ONPs in parallel with wt-iPSCs (Fig. 2A). Note that the hi-iPSCs underwent the same morphological changes as the control cells. At the end of the differentiation, both wt-iPSC-derived ONPs (wt-ONP) and hi-iPSC-derived ONPs (hi-ONP) exhibited protein expression of the neuronal markers Nestin, β-III Tubulin, and neurogenic differentiation 1 (NeuroD1). Both wt-ONPs and hi-ONPs also showed protein expression of the otic lineage markers PAX-8, GATA-3, EYA-1, and PAX-2 (Fig. 2B). Moreover, the surface expression of HLA Class I molecules was reduced in hi-ONPs when compared to the wild-type cells (Fig. 2C, D). Conversely, the surface expression of PD-L1 and HLA-G gradually decreased throughout differentiation (Additional file 1: Fig. S3).

### More hi-ONPs were observed in the cochlea after transplantation into immunocompetent mice

**Fig. 3:**
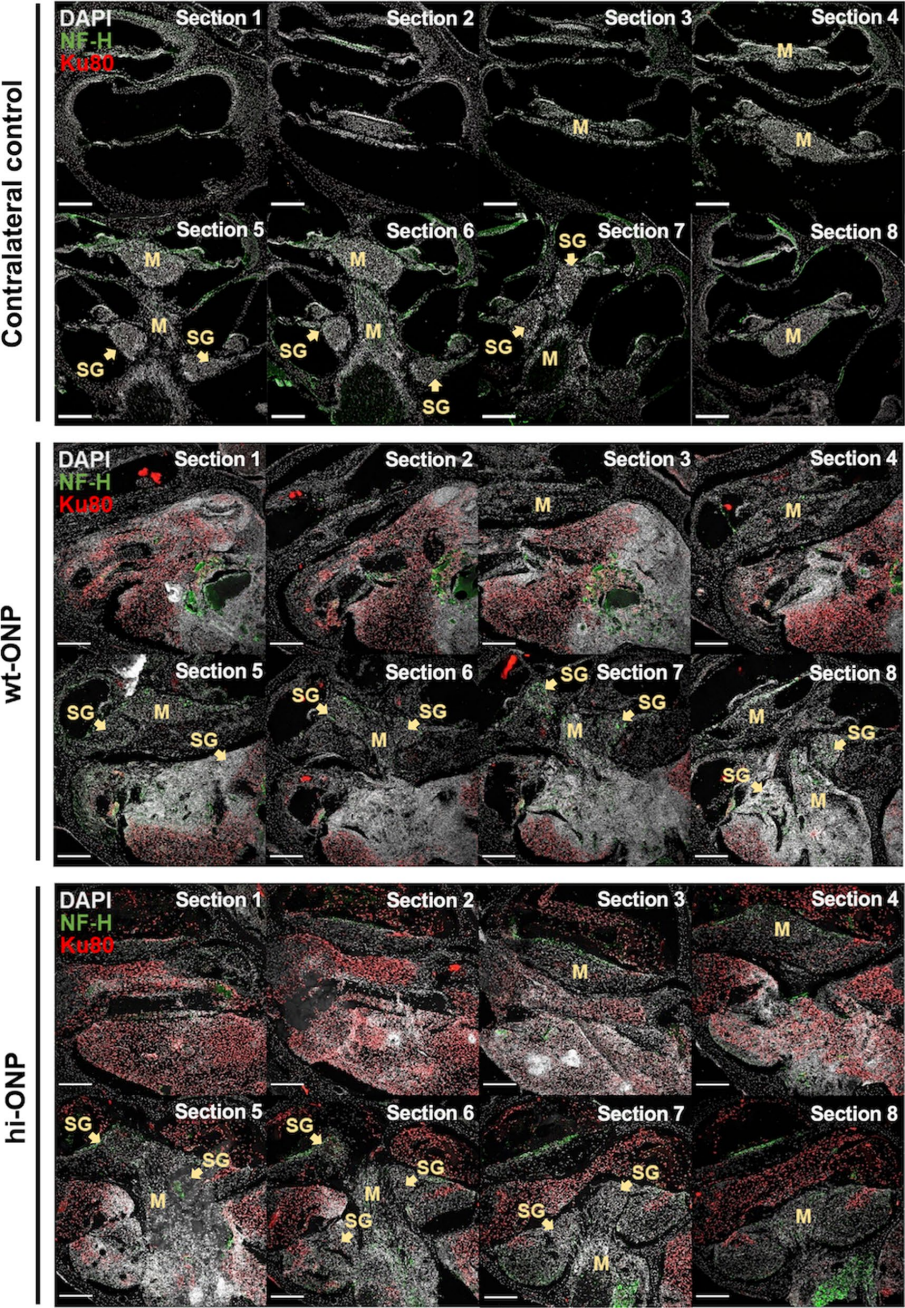
Localization of iPSC-derived ONPs 10 days following intracochlear transplantation. Representative confocal microscopy micrographs of eight serial sections from a contralateral control cochlea (top panel), a cochlea transplanted with wild-type ONPs (middle) and cochlea transplanted with hypoimmunogenic ONPs (bottom). The donor cells (DAPI^+^/Ku80^+^) are evidenced in red. Bars: 200 μm. NF-H: Neurofilament heavy chain; SG: Spiral Ganglion; M: Modiolus

To facilitate the inner ear transplantation through the round window, wt-ONPs and hi-ONPs were initially arranged in three-dimensional spheroids (Additional file 1: Fig. S4A), as previously described [18, 19]. Spheroids were transplanted into the left cochlea, while the right cochlea served as a control. Ten days after transplantation (Additional file 1: Fig. S4B), animals were euthanized, and both cochleae were dissected and cryoembedded. Then, mid-modiolar cryosections were prepared (as schematized in Additional file 1: Fig. S5) and immunoreacted with an antibody against the human nuclei marker Ku80, to localize the transplanted wt-ONPs or hi-ONPs. The donor cells, *i.e.,* positively stained for both DAPI and Ku80, are evidenced in red in Figs. 3, Additional file 1: Figs. S4C, S6, and S7. These DAPI^+^/Ku80^+^ signals were absent from the contralateral control cochlea (Fig. 3, top panel).

**Fig. 4:**
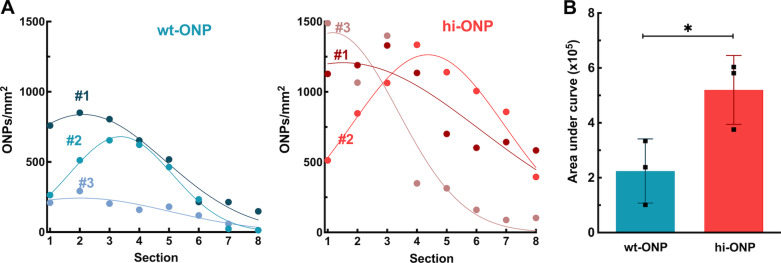
Quantification of iPSC-derived ONPs 10 days following intracochlear transplantation.** A** Distribution of ONP count/mm^2^ values of eight serial mid-modiolar sections, starting from the insertion region (Section 1), going toward the modiolus (Section 8). The area under curve values, which estimate the amount of ONPs per cochlear volume, are presented in (**B**). Bars represent means ± standard deviation from three independent experiments. *p < 0.05

The ONP counts in eight serial sections, 90 μm apart, are presented in Fig. 4A. Note that the animals transplanted with hi-ONPs (right panel, in red) had higher ONP/mm^2^ values than those transplanted with wt-ONPs (left panel, in blue). In fact, the area under the curve values—representing the number of donor cells per cochlear volume—were significantly higher in the hi-ONP group (Fig. 4B). Additionally, since the ONP number/mm^2^ values were normally distributed, we performed a second analysis in which the curves were aligned by the maximum value and grouped (Additional file 1: Fig. S8). The data indicate that animals transplanted with hi-ONPs have significantly more ONPs/mm^2^ in initial sections when compared to the control group. Altogether, these data suggest that ONPs with lower immunogenicity have better survival rates following transplantation into the inner ear of immunocompetent mice.

## Discussion

The establishment of induced pluripotent stem cell (iPSC) technology opened a new avenue for personalized medicine, in which patient-derived stem cells and their derivatives could be leveraged to treat a myriad of conditions. Despite this promising potential, such an approach is currently limited to small-scale clinical applications, given its laborious, time-consuming, and costly processing [16, 20]. In view of this, the generation of transplantable stem cells from allogeneic sources has become more attractive, since it is industrially scalable, economically feasible, and available in acute medical situations [20]. The success of allogeneic cell therapies, however, depends on the ability to avoid the clearance of donor cells by the host’s immune system. Here, we demonstrate the feasibility of this goal in the context of inner ear regeneration, by deriving transplantable ONPs from human iPSCs that lack HLAs, which showed improved engraftment in the inner ear of a murine host over the period when most exogenous cells are known to be cleared [21–23].

The cochlea was long thought to lack immune activity. However, it was recently reported that a population of macrophages (CD163^+^, CD68^+^_,_ IBA1^+^, and HLA Class II^+^) reside in the connective tissues of the lateral wall, in the modiolus, in the spiral ganglion, and in the Organ of Corti [9, 10]. This population increases in response to local surgical stress and to damage induced by noise and ototoxic drugs, for example [9, 24–26]. Importantly, the cochlear macrophages were observed close to both CD4^+^ and CD8^+^ T lymphocytes, which suggests that they can present antigens and initiate a local adaptative immune response [10].

This fact is particularly relevant in the context of stem cell-based replacement therapies for SNHL, since allogeneic immune rejection to transplanted cells is primarily mediated by T cell-dependent immune responses [9]. In view of this, the hi-iPSCs used in the present study were genetically engineered to evade T cell recognition and response by two distinct mechanisms. The first is the prevention of HLA mismatches between donor and recipient, which triggers T cell activation [13], by disrupting HLA class Ia and II genes in the hi-iPSCs (Figs. 1 and 2). The second is an approach inspired by naturally occurring immune tolerance mechanisms, which is the overexpression of the immune checkpoint proteins HLA-G and PD-L1, factors that inhibit T cell cytolytic action [27, 28]. Although HLA-G and PD-L1 overexpression gradually decreased upon differentiation toward ONP (Additional file 1: Fig. S3), our data suggest that the lack of HLA Class Ia surface expression already leads to enhanced survival of hi-iPSC derivatives.

In accordance with our findings, other reports had previously shown that some genetically modified PSC lines do not sustain transgene expression upon differentiation toward neuronal lines [29, 30]. Although hi-ONPs presented increased survival rates albeit low HLA-G and PD-L1 expression, we hypothesize that their additional role on preventing missing self-response would further prolong hi-ONPs residence time in the inner ear. Future studies will also address strategies to overexpress and sustain the expression of genes that play roles in immune evasion, such as CD47 [15, 16].

Lastly, some safety concerns need to be addressed prior to translating the Universal Donor Cell technology into clinical practice. These include the possibility of the HLA genes ablation to interfere with the clearance of some donor cells that may become apoptotic or malignant during the delivery process [13]. In this case, insertion of a ‘suicide’ gene that is inducible upon cell apoptosis/proliferation may be necessary as quality control.

In summary, in the present work, we demonstrated the feasibility of a strategy designed to confer donor cells protection against cochlear immune responses, thereby enhancing their survival following transplantation into the inner ear. By leveraging a hypoimmunogenic iPSC cell line, otic neural progenitors (ONP) with reduced immunogenicity were generated, which displayed an increased in vivo residence time in the inner ear. Importantly, this strategy opens new possibilities to improve current stem cell-based replacement therapies for inner ear disorders, including SNHL.

## Experimental section

### Hypoimmunogenic ONPs generation

The hypoimmunogenic iPSCs cell line was generated by HEALIOS K.K. (manuscript in preparation). In brief, the HLA-A, HLA-B, HLA-C and RFXANK genes have been sequentially knocked out in an iPSC line (Lonza) using a ribonucleoprotein complex consisting of Alt-R® S.p. HiFi Cas9 Nuclease (Integrated DNA Technologies, Inc.) and 2-piece TrueGuide Synthetic gRNA (Thermo Fisher Scientific Inc.), following the manufacturer instructions. Then, HLA-G, PD-L1, PD-L2 and iCasp9 [31] have been ectopically expressed under the control of human EF- 1α promoter using PiggyBac system. After single-cell cloning by serial dilution, the single iPSC clone having biallelic frame shift mutation at the 4 gene loci as well as expressing all the 4 factors was selected.

Differentiation of hi-iPSCs to hi-ONPs was performed following our already established protocol [32]. See also details in Additional file 1: Table S1. Flow cytometry analysis, immunocytochemistry, and reverse transcription followed by quantitative polymerase chain reaction (RT-qPCR) were performed to confirm genomic modifications as well as ONPs differentiation. Further details are provided in SI methods.

### Characterization of hypoimmunogenic iPSCs and ONPs

Hi-iPSC pluripotency was analyzed by evaluating mRNA levels of *NANOG*, *POU5F1*, and *SOX2* by RT-qPCR and also by immunocytochemistry of these markers (See details in Additional file 1: Tables S2 and S3). RT-qPCR was also performed to compare mRNA levels of *HLA-G* and *PD-L1* genes between hi-iPSCs and wt-iPSCs. Lack of surface expression of HLA-A, HLA-B, and HLA-C, and overexpression of HLA-G and PD-L1 in hi-iPSCs and their derivatives were analyzed by flow cytometry (See details in Additional file 1: Table S4). Successful differentiation toward ONP stage was evaluated by immunocytochemistry. The detailed procedures are described in SI methods.

### Assessment of hi-ONPs survival following intracochlear transplantation

The Institutional Animal Care and Use Committee (IACUC) of the Northwestern University Feinberg School of Medicine approved our experimental procedures (IACUC Protocol number: IS00011516). The US Army Animal Care and Use Review Office also reviewed and approved the protocol. We followed the ARRIVE guidelines for the reporting of animal experiments. Both male (*N* = 17) and female (*N* = 22) C57BL/6 mice (3–6 months old) were used as ONP recipients. The mice were needed to explore different time points and different types of hydrogels.The mice were randomly selected for each allocated experimental condition; however, experimenters were aware of whether a mouse was in the control group or not, as we used the right side of the cochlea as a control to reduce the number of mice required for the study. The mice were chosen from a different cage. However, sometimes, we used two mice in the same cage to reduce the number. The exclusion criteria for this study are as follows 1) middle ear infection, 2) poor growth, and 3) postop complications (euthanized before the predetermined time point).

Seven days before transplantation, wt-ONPs and hi-ONPs were arranged in three-dimensional spheroids, as previously described [18, 19]. The spheroids were then inserted into the left cochlea of immunocompetent C57BL/6 mice, through the round window (Additional file 1: Fig. S4B). The right cochlea served as a control. After 10 days, mice were euthanized for sample collection for post-mortem examination, such as histological analyses. For these mice used for histological analyses, thoracotomy under deep anesthesia was used as the primary method, and exsanguination was used as the secondary method. Mice were also euthanized in case of  pain or distress. For these, CO_2_ inhalation was used as the primary method, and cervical dislocation and decapitation were used as the secondary method. These methods are consistent with the AVMA Guidelines on Euthanasia (AVMA 2013 or later editions). After fixation and decalcification, specimens were mounted in optimal cutting temperature compound (OCT), placing the round window (ONP insertion point) facing up. Transplanted and control cochleae were then entirely cryosectioned into mid-modiolar 30 μm sections. Every 3rd section was collected onto microscope slides (Additional file 1: Fig. S5) and immunoreacted with a rabbit monoclonal anti-human Ku80 antibody to track the donor cells. The quantification of engrafted ONPs was performed on eight serial sections per mouse, spaced 90 µm apart. The detailed donor cell quantification procedures are described in SI methods.

### Statistical analysis

The sample size was determined using G*Power version 3.1.9.7 [33]. Using a *priori* power analysis, sample size N was computed as a function of the required power level (1 − *β*), the pre-specified significance level *α*, and the population effect size to be detected with probability (1 − *β*). In this study, we set *α* = 0.05, power (1 − *β*) = 0.95, and effect size *d* = 0.2 (small effect) suggested by Coen [34]. In comparison of hi-ONPs with wt-ONPs at the 10-day timepoint, we used *N* = 10.

Statistical data analyses were performed in GraphPad Prism version 9.0. First, the normality of the data was tested using the Shapiro-Wilk test; then, the ROUT test was performed to identify outliers. If data were normally distributed, Welch's t*-*test*,* Brown-Forsythe and Welch analysis of variance (ANOVA) (followed by Dunnett T3 post hoc test), or two-way ANOVA (followed by Bonferroni's post hoc test) were used. Differences were considered statistically significant at *P* < 0.05.

## Supplementary Information

**Additional file 1**. Supplemental information. **Section 1: Supplementary methods. 1.1. **Differentiation of hi-iPSCs toward otic neural progenitors. **1.2.** Quantitative RT-qPCR. **1.3.** Immunocytochemistry. **1.4. **Flow cytometry analysis. **1.5.** Generation of ONP spheroids for transplantation. **1.6.** Intracochlear transplantation of ONPs through round window. **1.7.** Immunohistochemistry and donor cell quantification; **Section 2: Supplementary figures**. **Figure S1. **Overexpression of HLA-G and PD-L1 genes in hypoimmunogenic iPSCs. **Figure S2. **G-banded karyotyping of hypoimmunogenic iPSCs. **Figure S3. **Surface expression of HLA-G and PD-L1 transgenes diminishes in hypoimmunogenic cells during differentiation from iPSC to ONP stage. **Figure S4. **Intratympanic transplantation of ONP spheroids. **Figure S5. **Representation of the mid-modiolar cryosections used for quantification of donor cells. **Figure S6. **Intracochlear transplantation of wild-type iPSC–derived ONP spheroids (wt-ONP). **Figure S7. **Intracochlear transplantation of hypoimmunogenic iPSC–derived ONP spheroids (hi-ONP). **Figure S8. **Distribution of ONP count/mm² values.

## Data Availability

The datasets analyzed during the current study will be provided by the corresponding author upon reasonable request.

## Abbreviations

HC: Hair cells
hi-iPSC: Hypoimmunogenic induced pluripotent stem cell
HLA: Human leukocyte antigen
ONP: Otic neural progenitor
PD-L1: Programmed death ligand-1
PSC: Pluripotent stem cells
SGN: Spiral ganglion neurons
SNHL: Sensorineural hearing loss
wt-iPSC: Wild-type induced pluripotent stem cell
