# Supplementary material for: Enhanced survival of hypoimmunogenic otic progenitors following intracochlear xenotransplantation: repercussions for stem cell therapy in hearing loss models
Source: Stem Cell Res Ther. 2023 Apr 12;14:83. doi: 10.1186/s13287-023-03304-9 (PMC10099643; doi:10.1186/s13287-023-03304-9)
Supplement: Supplementary file 1 — Additional file 1. Supplemental information. Section 1: Supplementary methods. 1.1. Differentiation of hi-iPSCs toward otic neural progenitors. 1.2. Quantitative RT-qPCR. 1.3. Immunocytochemistry. 1.4. Flow cytometry analysis. 1.5. Generation of ONP spheroids for transplantation. 1.6. Intracochlear transplantation of ONPs through round window. 1.7. Immunohistochemistry and donor cell quantification; Section 2: Supplementary figures. Figure S1. Overexpression of HLA-G and PD-L1 genes in hypoimmunogenic iPSCs. Figure S2. G-banded karyotyping of hypoimmunogenic iPSCs. Figure S3. Surface expression of HLA-G and PD-L1 transgenes diminishes in hypoimmunogenic cells during differentiation from iPSC to ONP stage. Figure S4. Intratympanic transplantation of ONP spheroids. Figure S5. Representation of the mid-modiolar cryosections used for quantification of donor cells. Figure S6. Intracochlear transplantation of wild-type iPSC–derived ONP spheroids (wt-ONP). Figure S7. Intracochlear transplantation of hypoimmunogenic iPSC–derived ONP spheroids (hi-ONP). Figure S8. Distribution of ONP count/mm² values. [file 13287_2023_3304_MOESM1_ESM.pdf]

Supplemental Information

Enhanced survival of hypoimmunogenic otic progenitors following intracochlear xenotransplantation – Repercussions for stem cell therapy in hearing loss models

Luisa Andrade da Silva, Rachel Heuer, Christian Roque, Tammy McGuire, Tomonori Hosoya, Hironobu Kimura, Kouichi Tamura, Akihiro J. Matsuoka

1. Supplementary methods

1.1. Differentiation of hi-iPSCs towards otic neural progenitors

To differentiate Wt-iPSCs (passages 20–26) and hi-iPSCs (passages 43-49) into ONPs, some modifications were made to our previously established protocol. First, undifferentiatied iPSCs were cultured in StemFit Basic02 media (Ajinomoto CO., INC, Tokyo, Japan) on iMatrix-511TM (Nippi, Tokyo, Japan). After magnetic purification of undifferentiated iPSCs using an anti-TRA-1-60 microbeads kit, LS Columns, and a MidiMACS Separator (Miltenyi Biotec, Bergisch Gladbach, Germany), cells were seeded into a chemically defined medium containing: 40% DMEM/F12 medium (Gibco, Gaithersburg, MD, USA), 40% Neurobasal medium (Gibco) and 20% StemFit for Differentiation supplement (Ajinomoto).

Then, a stepwise series of small signaling molecules and growth factors were added to promote iPSC differentiation towards a late-stage ONP lineage, as presented below. The human recombinant basic fibroblast growth factor (bFGF, Catalog No. 78003. StemCell Technologies, Vancouver, BC, Canada) was used in all differentiation steps and does not appear in the following table.

Table S1. Materials used for iPSC differentiation to otic neural progenitors

Differentiation stage	Small molecules/Growth factors	Manufacturer/ Catalog No.
1. Nonneuronal ectoderm	Human recombinant bone morphogenetic protein 4 (BMP4)	StemCell Technologies/78211
	SB431542	Reprocell/04-0010-05
2. Preplacodal ectoderm	LDN-193189	Reprocell/ 04-0074-02
	IWP-2	StemCell Technologies/ 72122
3. Early/mid-stage ONPs	CHIR-99021	StemCell Tecnologies/72052
	Human recombinant insulin-like growth factor (IGF)-I	StemCell Technologies/ 78142
4. Late-stage ONPs	Human recombinant epidermal growth factor (EGF)	StemCell Technologies/78006
	All-Trans Retinoic Acid (ATRA)	StemCell Technologies/72262
	Human recombinant Sonic Hedgehog (SHH)	R&D systems/ 1845-SH-025

1.2. Quantitative RT-qPCR

Cells were initially lysed for RNA extraction through the ReliaPrep RNAMiniprep System (Promega Corporation, Madison, Wisconsin) in accordance with manufacturer recommendations. The total RNA concentration and purity was measured by spectrophotometry in a Nanodrop ND-1000 system (Thermo Fisher Scientific, Waltham, Massachusetts). A260/A230 and A260/A280 ratios approximately equal to 2 were considered ideal for RNA purity.

First-strand cDNA was synthesized from 1μg RNA using a high-capacity cDNA reverse transcription kit (Thermo Fisher Scientific). Relative mRNA levels were measured by SYBR Green detection (PowerUp™ SYBR™ Green Master Mix, Thermo Fisher) in a CFX Connect Real-Time PCR Detection System (Bio-Rad Laboratories, Hercules, CA, USA). All samples were measured in triplicate. The relative level of each gene was calculated as the ratio of the study gene to the control gene (GADPH). The primers used for RT-qPCR and respective 5'-3' sequences are presented below:

Table S2. Primer sequences for RT-qPCR analysis

Gene	Forward primer	Reverse primer
GADPH	ACCACAGTCCATGCCATCAC	TCCACCACCCTGTTGCTGTA
HLA-G	TTGGGAAGAGGAGACACGGAACA	AGGTCGCAGCCAATCATCCAC
NANOG	CAGCCCCGATTCTTCCACCAGTCCC	CGGAAGATTCCCAGTCGGGTTCACC
OCT 4/ POU5F1	GACAGGGGGAGGGGAGGAGCTAGG	CTTCCCTCCAACCAGTTGCCCCAAAC
PD-L1	TGCCGACTACAAGCGAATTACTG	CTGCTTGTCCAGATGACTTCGG
SOX 2	GGGAAATGGGAGGGGTGCAAAAGAGG	TTGCGTGAGTGTGGATGGGATTGGTG

1.3. Immunocytochemistry

Late-stage ONPs and iPSCs were initially seeded on matrigel-coated coverslips and cultured for at least 48 hours prior to staining. Cells were first rinsed once with PBS, fixed in 4% (w/v) paraformaldehyde in PBS for 15 minutes, and then washed twice with PBS for 5 minutes. Cell permeabilization was performed using 0.3% Triton X-100 (Sigma-Aldrich, St. Louis, MO, USA) in PBS for 30 minutes at room temperature, under agitation, and washed with PBS three times for 5 minutes each. Blocking was performed using 10% BSA (Sigma-Aldrich, St. Louis, MO, USA) and 0.1% Triton X-100 in PBS for 1 hour at room temperature, under agitation.

Immunoreaction with primary antibodies was performed overnight at 4°C, under agitation. Cells were subsequently incubated for 1 hour with fluorescence-labeled secondary antibodies and counterstained with DAPI (DAPI; Cat No. D1306; Thermo Fisher Scientific, Waltham, MA, USA). Last, the coverslips were washed three times with PBS and mounted using ProLong® Gold Antifade reagent (Invitrogen, Carlsbad, CA, USA). Images were acquired using Nikon A1R confocal microscope (Nikon, Tokyo, Japan). The following antibodies were used for immunocytochemistry analysis:

Table S3. Antibodies used for immunocytochemistry analysis

Primary antibody	Manufacturer	Catalog No.	Dilution
Anti-β III Tubulin	Abcam	Ab18207	1:100
Anti-EYA 1	Sigma-Aldrich	SAB2501395	1:100
Anti-GATA 3	R&D biosystems	MAB6330	1:100
Anti-NANOG	Pierce	PAS-18618	1:100
Anti-Nestin	Abcam	Ab22035	1:100
Anti-NeuroD1	Abcam	Ab60704	1:100
Anti-OCT 4/POU5F1	Santa Cruz	Sc-9081	1:100
Anti-PAX 2	Abcam	Ab79389	1:100
Anti-PAX 8	Abcam	Ab13611	1:100
Anti-SOX 2	Santa Cruz	Sc-17320	1:100
**Secondary antibody**	**Manufacturer**	**Catalog No.**	**Dilution**
Alexa Fluor 488nm Donkey anti-goat	Thermo Fisher Scientific	A11055	1:500
Alexa Fluor 594nm Donkey anti-mouse	Thermo Fisher Scientific	A21203	1:500
Alexa Fluor 647nm Donkey anti-rabbit	Thermo Fisher Scientific	A31573	1:500

1.4. Flow cytometry analysis

Late stage ONPs and iPSCs were initially pre-incubated in ice-cold 5% BSA in PBS, for 10 minutes, to avoid non-specific protein binding. They were then centrifuged and resuspended in eBioscienceTM Flow Cytometry Staining Buffer ($2x10^5$ cells in a 49μl volume) and immunoreacted with fluorochrome conjugated-primary antibodies (1:50 dilution), at room temperature for 1 hour at dark. Last, cells were washed with PBS and fixed in 1% PFA in PBS. Cell suspensions were run in a LSRFortessa™ Flow Cytometer (BD Biosciences, San Jose, CA, USA) and data were analyzed using FlowJo v10.6.1 software (FlowJO, LLC, Ashland, Oregon, USA). The following antibodies were used for flow cytometry analysis:

Table S4. Antibodies used for flow cytometry analysis

Conjugated primary antibody	Manufacturer	Catalog No.
FITC Anti-HLA-A2	Biolegend	343303
APC Anti-HLA Bw4	Miltenyi Biotec	130-103-918
PE Anti-HLA C	BD Biosciences	566372
APC Anti-HLA-G	Biolegend	335909
APC Anti-PD-L1	eBioscience	17-5983-41

1.5. Generation of ONP spheroids for transplantation

Upon reaching the late ONP stage, wt-ONPs and hi-ONPs were passaged to a 96-well Clear Round Bottom Ultra-Low Attachment Microplate® (U-bottom, #7007; Corning Life Science, Tewksbury, MA), and seeded at $5x10^4$ cells/well. The plate was then centrifuged at 201 relative centrifugal force for 10 min. The three-dimensional culture system was maintained for 7 days.

1.6. Intracochlear transplantation of ONPs through round window

Meloxicam (2 mg/kg, subcutaneous injection) was given prior to surgery to minimize respiratory distress; follow-up doses (1 mg/kg/day) were given for three days. Anesthesia was induced with isoflurane at 3–4% (reduced to 1–2% after induction) and was delivered with oxygen (0.2 L/min) and nitrous oxide (0.25 L/min) gas. The animals' heads were secured to a custom head-holder that delivered the anesthetic gas. Body temperature was maintained by a circulating water pad, and insensate fluid loss was replaced at 0.1 mL/10g (body weight). A post-auricular incision was made, and bone (caudal to the stylomastoid foramen) was removed with a surgical drill to reveal the round window. The round window membrane was then excised using a 30–33 G needle. Wt-iPSC-derived or hi-iPSC-derived ONP spheroids were delivered to the scala tympani with the aid of micropipettes. Following spheroid implantation, a nanofibrillar cellulose hydrogel solution (1% GrowDex-T, UPM Biomedicals, Helsinki, Finland) containing a brain-derived neurotrophic factor (BDNF) sustained release system (PODS®- hBDNF, Cell Guidance Systems, Cambridge, UK) were also delivered to the inner ear to create a supportive niche for the donor cells. Once the transplantation was completed, the round window was covered by a small piece of fascia and secured with Vet Bond™ adhesive (3M, Minneapolis, MN, USA). Muscle and skin were closed in layers, and animals were allowed to recover with thermal therapy. All transplantations were performed in the left cochlea, while the right cochlea served as a control.

Finally, confounders such as the order of treatments and measurements, animal/cage location were not controlled in this study.

1.7. Immunohistochemistry and donor cell quantification

10 days after intracochlear transplantation of ONPs, mice were euthanized, and left and right cochleae were dissected and fixed with 4% paraformaldehyde overnight at 4°C. For decalcification of the bone structure, the cochleae specimens were kept on 14% EDTA solution in PBS (pH 7.4), at 4°C, for 5-7 days. Then, cochleae were mounted in optimal cutting temperature compound (OCT), placing the round window region (ONP insertion point) facing up. Specimens were frozen and stored at −20 °C freezer. The entire cochlea was sliced into 30 µm slices on a Leica CM3050 S cryostat (Leica Inc., Nussloch, Germany). Every 3rd section was collected onto Superfrost™ Plus Microscope Slides (Fisherbrand, Cat # 12-550-15) for subsequent immunohistochemistry assay. The quantification of engrafted ONPs was performed on eight serial sections per mouse, spaced 90 µm apart (as schematized in figure S5).

The tissue sections were permeabilized with 0.25% (v/v) Triton X-100 for 30 min, blocked in 10% (v/v) normal goat serum, 5% (w/v) bovine serum albumin, and 0.1% Tween-20 in PBS for 1 h at room temperature (20–25 °C). Slices were incubated overnight with primary antibodies, diluted at blocking solution, at 4°C. Primary antibodies used were rabbit anti-human Ku80 (Cell Signaling Technology, 2180S, 1:100), and chicken Anti-Neurofilament Heavy (Millipore-sigma, AB5539, 1:200). Tissue sections were then rinsed three times with 0.1% Tween-20 in PBS before incubation with the secondary antibodies for 1 h at room temperature (20–25 °C). Alexa Fluor conjugated secondary antibodies used included Alexa Fluor 568nm Goat anti-rabbit (Thermo Fisher Scientific, Cat No. A-11011, 1:250), and Alexa Fluor 488 nm Goat anti-chicken (Thermo Fisher Scientific, Cat No. A-11039, 1:500). Nuclei were counterstained with DAPI. The contralateral cochlea, without transplanted ONPs, were used as controls.

Three dimensional images were generated through Z-stacks acquisition, using Nikon A1R confocal microscope (Nikon, Tokyo, Japan). We defined the engrafted cells as those which were double-positive for Ku80 and DAPI staining in the nuclei. To determine cells per area, the Image-J cell counter plugin was used to count the number of cells from a 10x objective image that included all the section, and this number was divided by the area of cochlear tissue to calculate cells/mm^2 values.

2. Supplementary figures

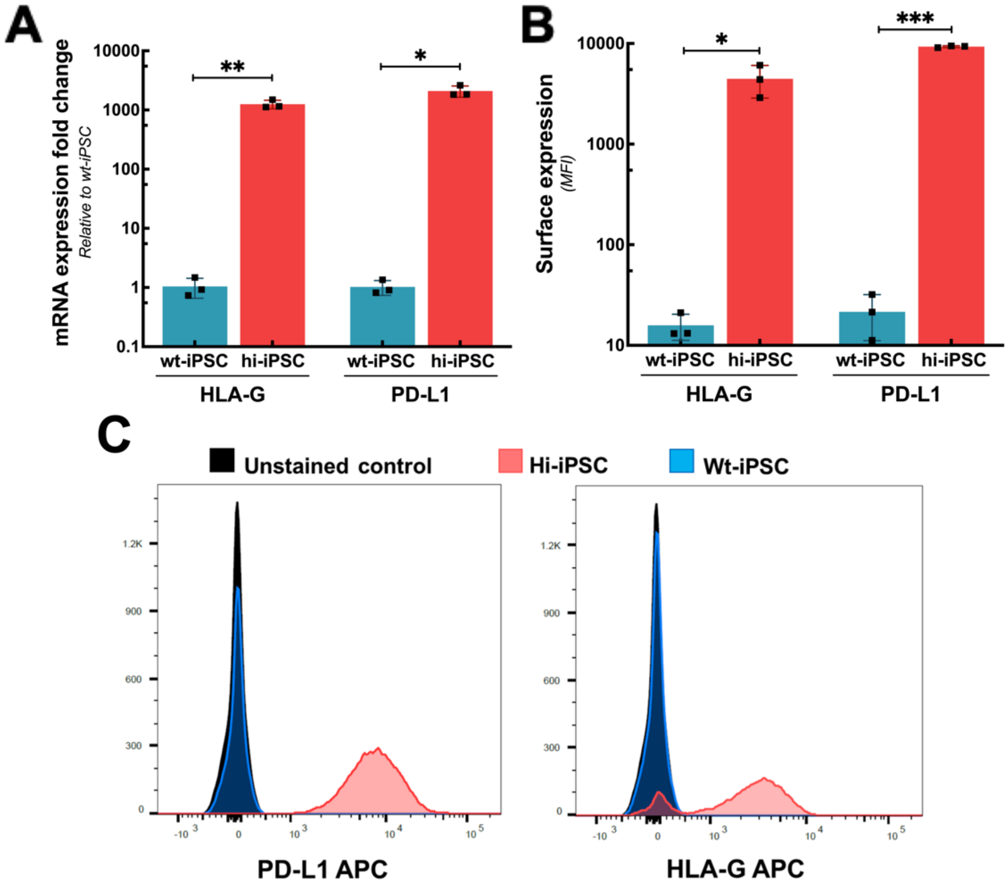

Figure S1. Overexpression of HLA-G and PD-L1 genes in hypoimmunogenic iPSCs. (A) Quantification of mRNA levels of the transgenes by RT-qPCR analysis. Data show the average and standard deviation of fold change values from three independent experiments. (*) $p < 0.05$, (**) $p < 0.01$. **(B-C)** Flow cytometry analysis. **(B)** Bars represent means ± standard deviation of mean fluorescent intensity values from three independent experiments. (*) $p < 0.05$, (***) $p < 0.001$. **(C)** Representative flow cytometry charts.

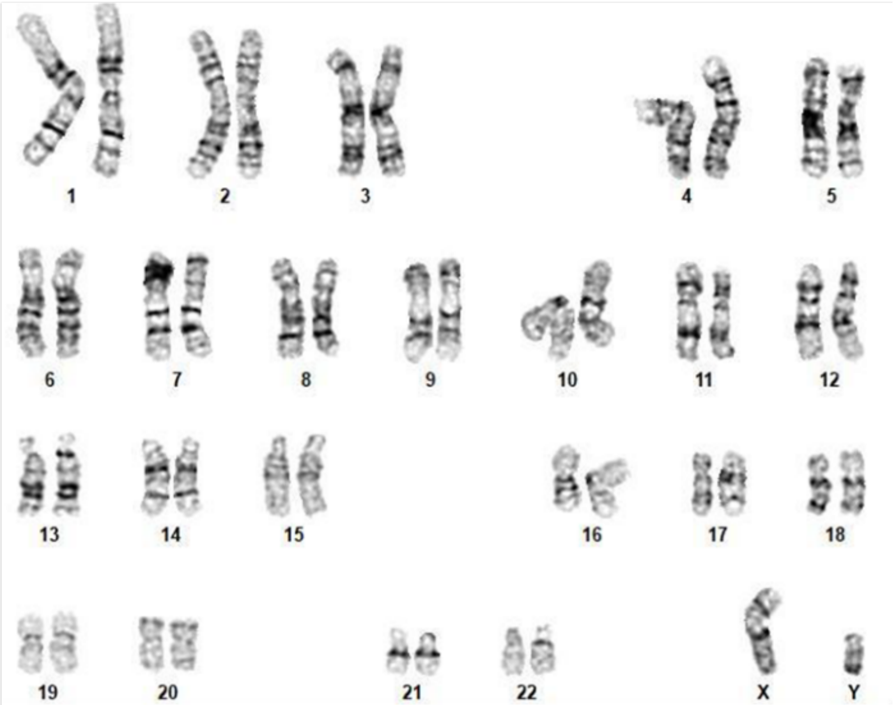

Figure S2. G-banded karyotyping of hypoimmunogenic iPSCs. Note that hi-iPSCs display normal karyotype after genomic engineering.

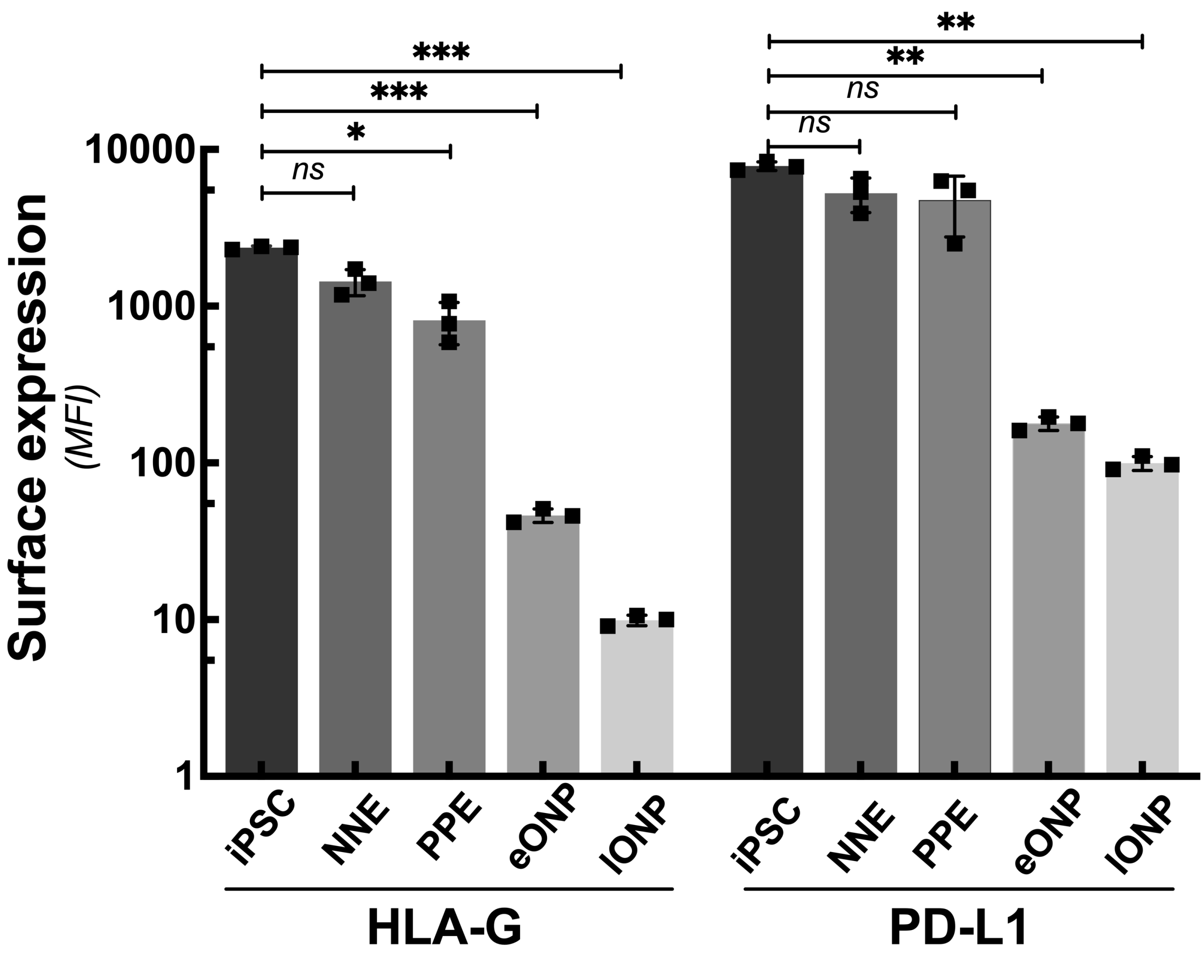

Figure S3. Surface expression of HLA-G and PD-L1 transgenes diminishes in hypoimmunogenic cells during differentiation from iPSC to ONP stage. Data show the average and standard deviation of mean fluorescent intensity values from three independent experiments. (*) $p < 0.05$, (**) $p < 0.01$, (***) $p < 0.001$.NNE: non-neuronal ectoderm; PPE: pre-placodal ectoderm; eONP: early-stage ONP; lONP: late-stage ONP.

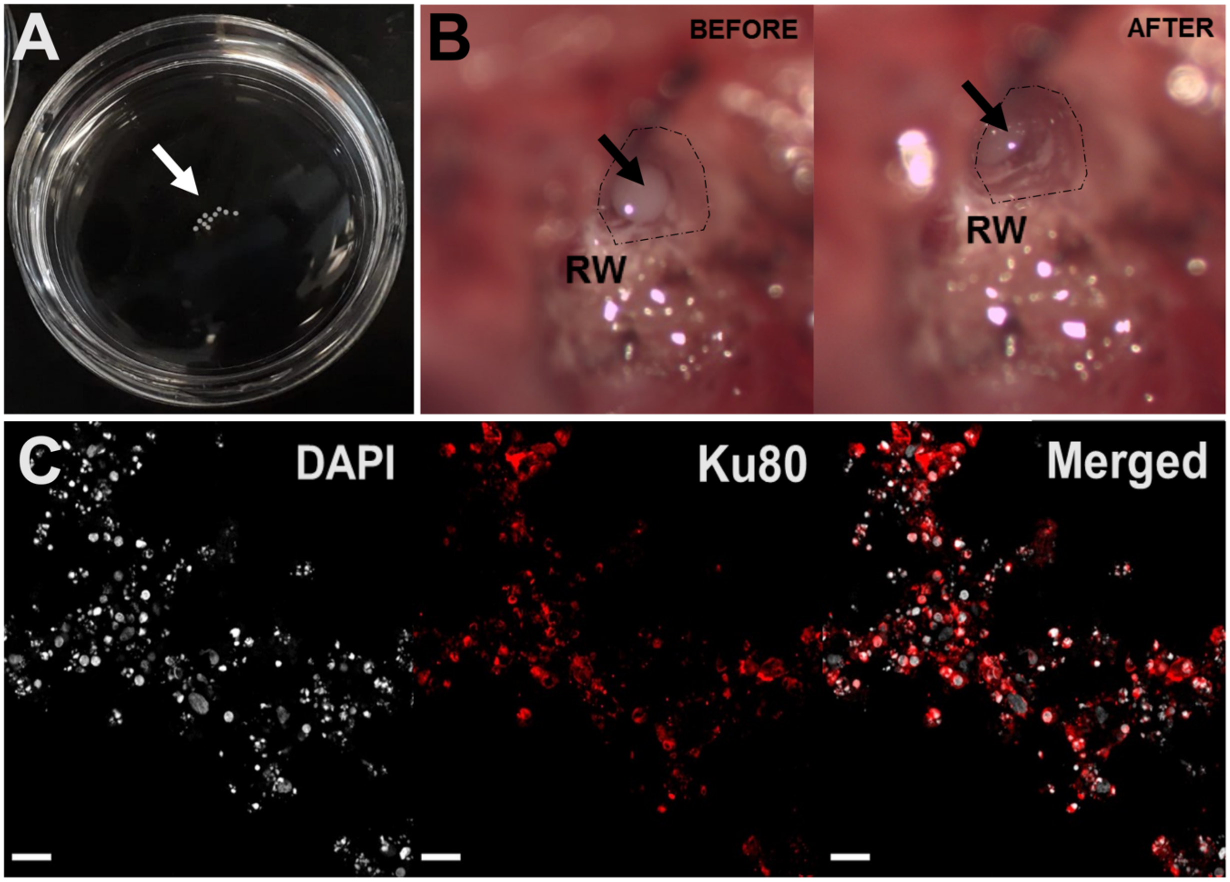

Figure S4. Intratympanic transplantation of ONP spheroids. ONPs were firstly arranged in spheroids (10^5 cells/spheroid) using ultra-low attachment plates (Corning) and kept in culture for 7 days. The generated spheroids were then transferred to a sterile 3.5mm dish **(A)**, for transplantation. Note the relative size of spheroids (**A**, arrow). **(B)** ONP spheroids (black arrows) were delivered to scala tympani through round window (RW). Photos show the moments before and after spheroid insertion in round window. **(C)** Spheroid immunofluorescence analysis. An anti-Human Ku80 antibody was initially tested in frozen-sliced ONP spheroids (10 μm thickness). Bars: 50 μm.

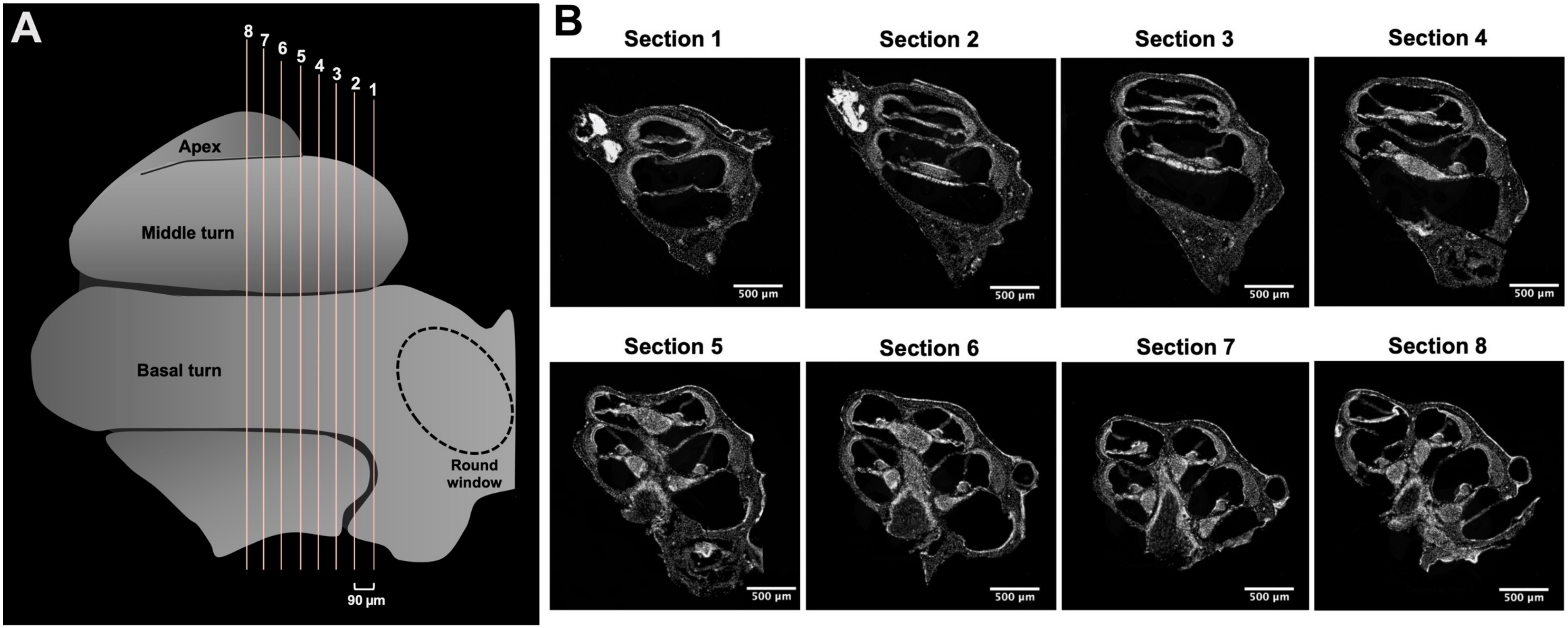

Figure S5. Representation of the mid-modiolar cryosections used for quantification of donor cells. (A) Sectioning started from the spheroid insertion point, *i.e.*, the round window, towards the modiolus. Eight serial sections, 90 µm apart, were used for posterior immunofluorescence analysis. **(B)** Representative confocal microscopy images of each section, obtained from a control cochlea without transplanted cells. Slices were stained with DAPI to evidence the structural features of each region. Bars: 500 µm.

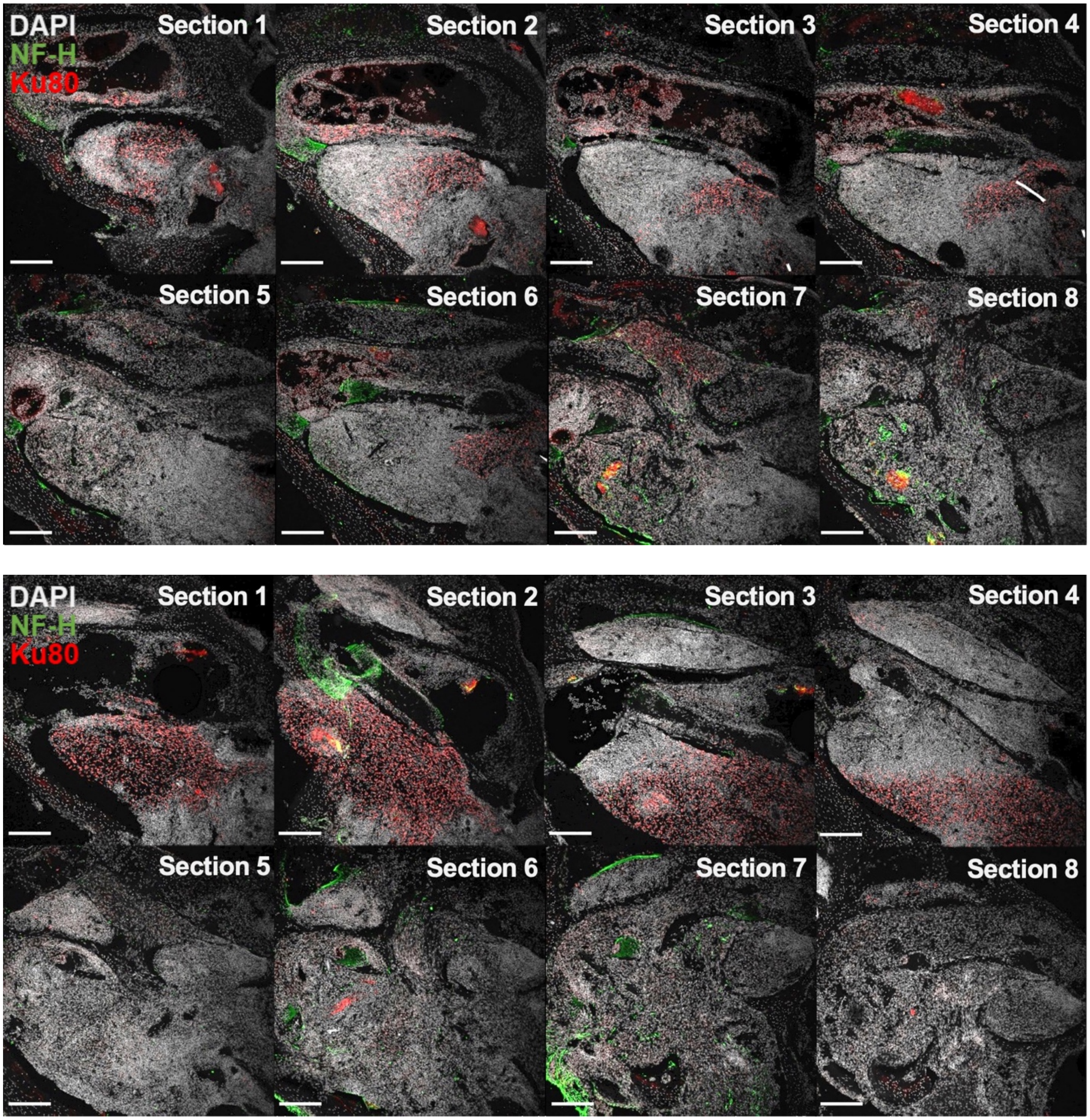

Figure S6. Intracochlear transplantation of wild-type iPSC–derived ONP spheroids (wt-ONP). Immunofluorescence analysis of cochlea 10 days after transplantation of wt-ONPs. The donor cells ($DAPI^+/Ku80^+$) are shown in red. Bars: 200 μm. NF-H: Neurofilament heavy chain.

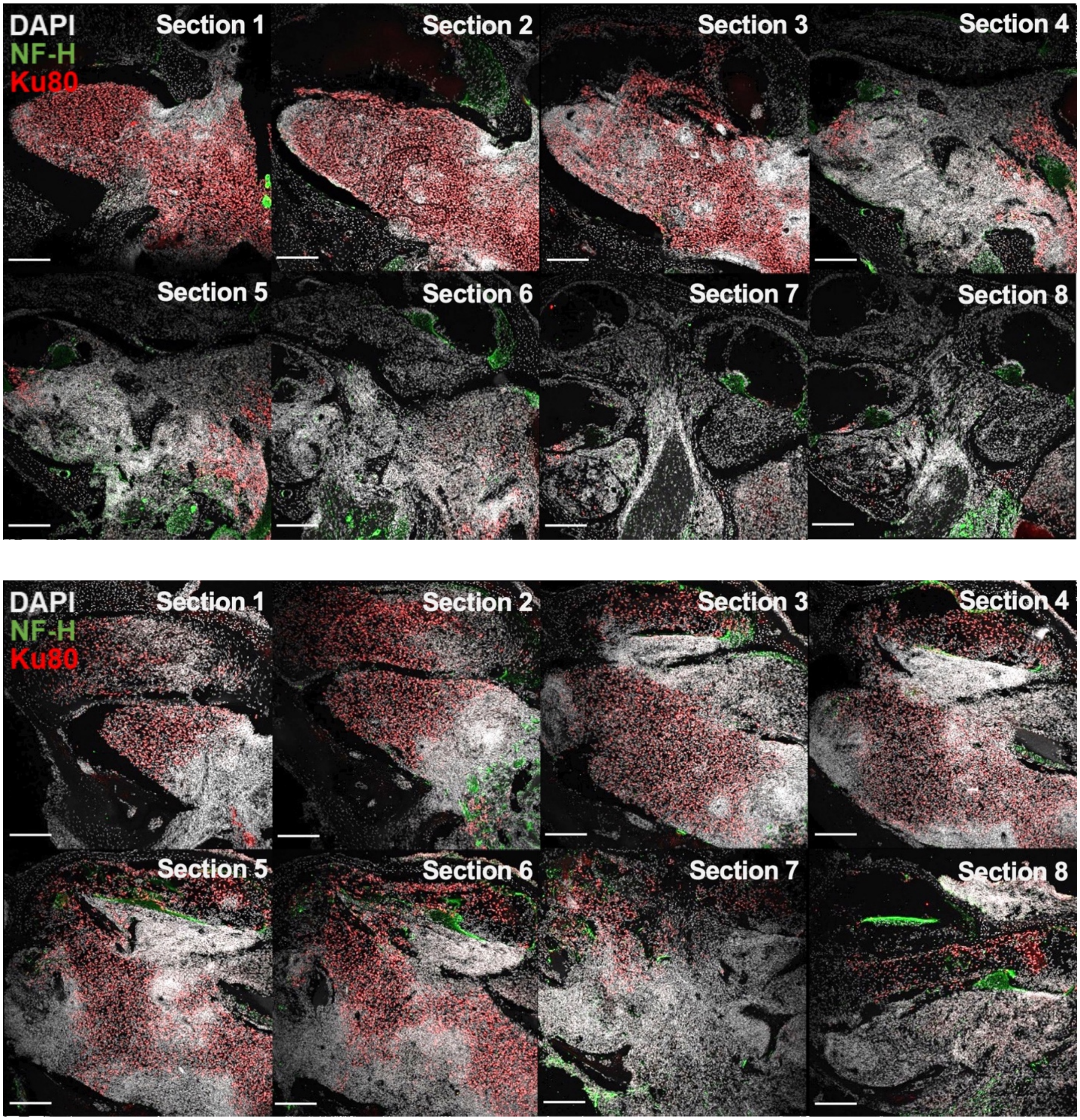

Figure S7. Intracochlear transplantation of hypoimmunogenic iPSC–derived ONP spheroids (hi-ONP). Immunofluorescence analysis of cochlea 10 days after transplantation of hi-ONPs. The donor cells (DAPI$^+$/Ku80$^+$) are shown in red. Bars: 200 µm. NF-H: Neurofilament heavy chain.

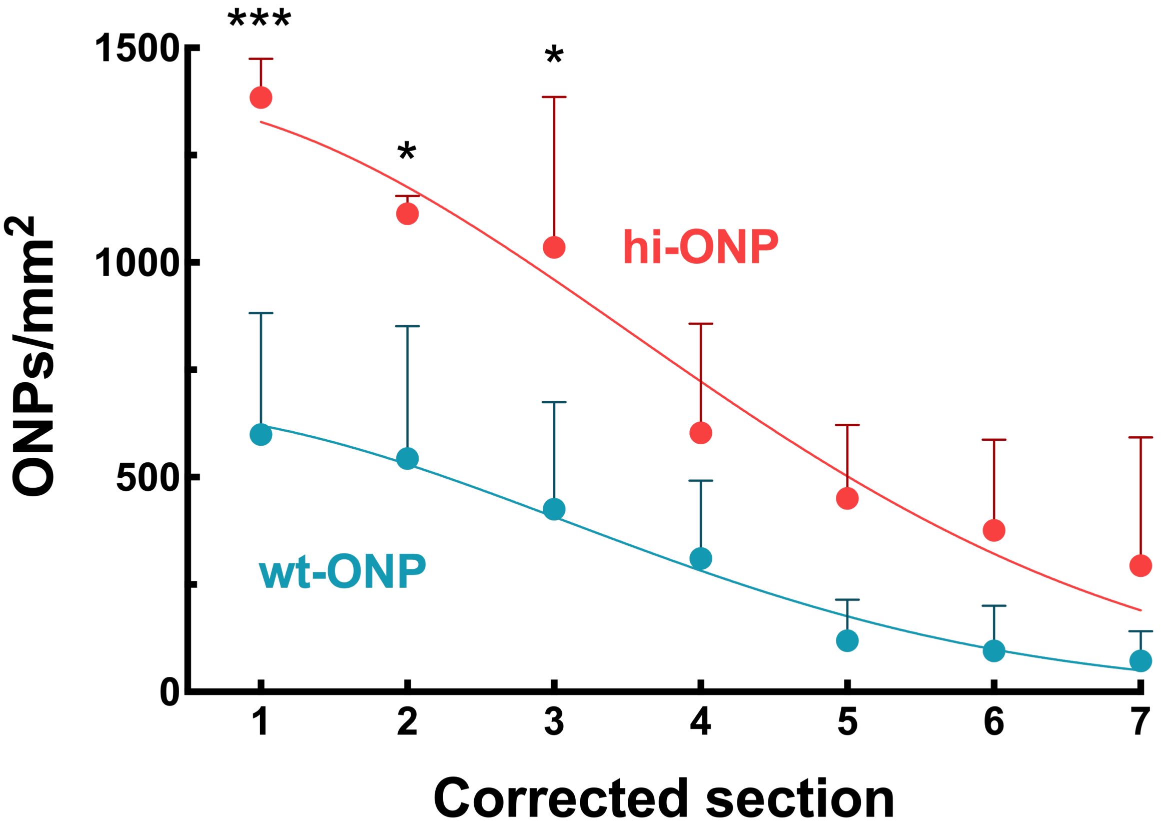

Figure S8. Distribution of ONP count/mm² values. The distribution curves were aligned by the maximum value and ONP count/mm² values were grouped. Dots represent means and whiskers represent standard deviation from three animals. (*) $p < 0.05$, (***) $p < 0.001$, when compared to *wt-ONP* group.
